# “Threshold‐crossing”: A Useful Way to Establish the Counterfactual in Clinical Trials?

**DOI:** 10.1002/cpt.515

**Published:** 2016-10-19

**Authors:** H‐G Eichler, B Bloechl‐Daum, P Bauer, F Bretz, J Brown, LV Hampson, P Honig, M Krams, H Leufkens, R Lim, MM Lumpkin, MJ Murphy, F Pignatti, M Posch, S Schneeweiss, M Trusheim, F Koenig

**Affiliations:** ^1^ European Medicines AgencyLondonUnited Kingdom; ^2^ Department of Clinical PharmacologyMedical University of ViennaViennaAustria; ^3^ Section for Medical Statistics, Center for Medical Statistics, Informatics, and Intelligent SystemsMedical University of ViennaViennaAustria; ^4^ NovartisBaselSwitzerland; ^5^ Harvard Medical School/Harvard Pilgrim Health Care InstituteHartfordConnecticutUSA; ^6^ Lancaster UniversityLancasterUnited Kingdom; ^7^ CollegevillePennsylvaniaUSA; ^8^ Janssen Pharmaceutical CompaniesRaritanNew JerseyUSA; ^9^ Medicines Evaluation Board, UtrechtUniversity of UtrechtUtrechtThe Netherlands; ^10^ Health CanadaOttawaOntarioCanada; ^11^ Bill and Melinda Gates FoundationSeattleWashingtonUSA; ^12^ Project Data SphereDurhamNorth CarolinaUSA; ^13^ Brigham and Women's Hospital and Harvard Medical SchoolBostonMassachusettsUSA; ^14^ MIT Sloan School of ManagementCambridgeMassachusettsUSA

## Abstract

A central question in the assessment of benefit/harm of new treatments is: how does the average outcome on the new treatment (the factual) compare to the average outcome had patients received no treatment or a different treatment known to be effective (the counterfactual)? Randomized controlled trials (RCTs) are the standard for comparing the factual with the counterfactual. Recent developments necessitate and enable a new way of determining the counterfactual for some new medicines. For select situations, we propose a new framework for evidence generation, which we call “threshold‐crossing.” This framework leverages the wealth of information that is becoming available from completed RCTs and from real world data sources. Relying on formalized procedures, information gleaned from these data is used to estimate the counterfactual, enabling efficacy assessment of new drugs. We propose future (research) activities to enable “threshold‐crossing” for carefully selected products and indications in which RCTs are not feasible.

## What is the counterfactual?

The human condition is an uncontrolled experiment. Socrates once advised a young man who asked whether he should get married: “Do as you wish, you will likely regret, no matter what you choose.” Why would the sage expect his friend to have postdecision blues? Whatever road the young man takes, he would certainly experience the consequences of his action (the factual) but there is no way he could learn the—possibly superior—consequences of the road not taken (the counterfactual). One might argue that the young man could still explore the counterfactual by marrying later in life or getting a divorce. However, this argument is flawed because the comparison between an early‐in‐life event with a late‐in‐life event is inadmissible; in modern research terms, the comparison is confounded. Alas, we shall rarely know the counterfactuals in our lives, the road not taken.

The assessment of the causal effects (benefits and harms) of any treatment revolves around the same question: how does the outcome of (test) treatment (the factual) compare to “what would have happened [if patients] had not received the test treatment or if they had received a different treatment known to be effective”^1^ (the counterfactual)? The question is asked by clinicians treating individual patients and by population‐level decision‐makers, including drug developers, regulators, health technology assessment (HTA) bodies, and payers of health care. However, the counterfactual outcomes of individual patients can rarely be observed. Decision‐makers must instead focus on comparing average population counterfactual outcomes between different interventions (which are estimable) to deduce causal effects on a population (see **Table** 1, 2, 3, 4, 5, 6, 7, 8, 9, 10, 11 for review of the concept of the counterfactual and how it underpins the definition of a causal treatment effect).

**Table 1:** Summary of key components relevant to the concept of the counterfactual and how it underpins the definition of a causal treatment effect

Term/concept	Description
Counterfactual	Suppose a patient may receive one of two treatments: an experimental drug E or a control C. Then patient i has two potential outcomes: their response if they receive treatment E, denoted by Y_i_(T_i_ = E), and their response if they receive treatment C, Y_i_(T_i_ = C). Only the outcome corresponding to the treatment actually received will be observed and, thus be factual, the other will remain counterfactual.
Causal effect	We often ask what is the effect on outcome Y of taking drug E (as opposed to drug C) keeping all other things equal?
Individual causal effect	The causal effect of drug E on individual i is measured by Y_i_(T_i_ = E) – Y_i_(T_i_ = C). However, individual causal effects are not identifiable because only one of a patient's potential outcomes can ever be observed (the factual but not the counterfactual).
Average causal effect	The average causal effect of drug E on outcome Y can be expressed as the difference between the mean counterfactual outcome that would be observed if all population members received treatment E and the mean outcome that would be observed if everyone received C.
RCT estimating causal effects	Under certain assumptions, average causal effects can be estimated from RCTs. Randomly assigning treatments to patients ensures that (at least under repeated sampling) treatment groups will be exchangeable in the sense that pairs of counterfactual outcomes (Y_i_(T_i_ = E), Y_i_(T_i_ = C)) will be distributed in the same way across patients in groups E and C. This implies that the average observed outcome in group C will equal the average counterfactual outcome that would be observed if all patients in group E had received drug C instead, and vice versa. Thus, the average causal effect of drug E can be estimated by comparing average outcomes across treatment arms.
Causation vs. association	Association is the phenomenon whereby two occurrences tend to be seen together, for example, higher response rates among contemporary patients on drug E than among historical controls. However, the risk of confounding means that association does not imply causation. Instead, the higher response rates on drug E may be attributable to a common cause linking both the treatment received and response. Examples include an imbalance in the baseline prognostic characteristics of patients, which may be a result of a drift in disease detection rates or improvements in patient management, or by fundamentally different patient populations being included in the studies.
Bias	The estimate of the average causal effect of drug E obtained from a direct comparison of average outcomes among contemporary experimental patients and historical controls will be biased if these groups are not exchangeable. Departures from exchangeability may arise due to a myriad of reasons including^2^: (a) confounding; (b) selection bias if the investigator intentionally cherry picks the historical control group to make the new treatment appear more effective than it really is; (c) external biases arising, for example, from the well‐known efficacy‐effectiveness gap^3^ if the historical cohort is taken from RWD assessing the effectiveness of control under the usual circumstances of health care practice, whereas the single‐arm trial will evaluate the efficacy of drug E in a controlled and idealized setting; and (d) internal biases inherent in the single‐arm trial due to a lack of blinding or allocation concealment.^4^, ^5^
Historical controls for estimating average causal effects	By comparing the outcome of a single‐arm trial with historical controls, we can estimate the average causal effect among contemporary patients of receiving drug E as opposed to drug C. Below, we outline a selection of techniques that could be used to control for confounding when comparing outcomes from a single‐arm trial with historical controls.
Multivariable regression	Obtain an estimate of the causal effect by fitting a regression model to the historical and contemporary data adjusting for all confounders.
Inverse probability of treatment weighting^6^, ^7^	Create exchangeable treatment groups by weighting each individual by their fitted probability of receiving the treatment they actually received given their baseline covariates. Estimate causal effects by fitting models using weighted least squares.
Propensity scores^8^	Conditional probability of an individual receiving drug E given their baseline covariates. Stratification^9^ or inverse probability weighting^7^ by estimated propensity scores can be used to deduce causal effects. However, using scores to create balanced treatment groups through patient matching can increase imbalance and bias.^10^
Instrumental variables^11^	Causal effects are identifiable if one can find a strong “instrument” associated with the outcome only through its association with the treatment received.

RCTs, randomized controlled trials; RWD, real world data.

How does the concept of the counterfactual underpin the definition of a causal treatment effect? Here, we review the current ways of estimating the counterfactual to enable the assessment of causal treatment effects. We reflect on how scientific and societal developments necessitate and enable a new way of determining the counterfactual for some new medicines. We then propose a new framework for evidence generation, which we will refer to as “threshold‐crossing.” Finally, we propose future research and other activities to enable a move toward threshold‐crossing for selected products and indications in which randomized controlled trials (RCTs) are not feasible.

For ease of presentation, throughout this paper we will refer to the average outcome on placebo/control corresponding to a group of patients who received a new experimental treatment simply as “the counterfactual.” For a comprehensive review of this topic, the reader is referred to Hernán and Robins.^12^

## The ascendancy of the RCT

The search for the counterfactual in medicine changed irreversibly with the introduction of the RCT. The concept of a concurrent control group had arisen earlier in history^13^ but the RCT era is generally considered^14^ to have begun in 1948 with the iconic RCT of streptomycin for pulmonary tuberculosis.^15^

The idea of the RCT was revolutionary in that it combined three important concepts into one methodology; each of which relates to one of the letters in the “R‐C‐T” acronym: (a) the recognition that the counterfactual for individual patients may never be known but the average counterfactual for groups of similar patients can be understood. Hence, the need to compare group‐to‐group averages rather than one patient to the next.^16^ This is the “T” in RCT. (Exceptions to this rule are clinical conditions that lend themselves to n‐of‐1 crossover trials; this study type will not be further considered here.) (b) The concept that the counterfactual for a group of patients treated with an (experimental) therapy can be determined by observing the outcome of interest in a control group that will remain untreated/receive placebo or be treated with another therapy. This relates to the “C” in RCT. (c) The recognition that the comparison between the experimental and the control groups needs to be unbiased and unconfounded in order to be meaningful (i.e., one needs to compare like‐with‐like).^13^ This goal is best achieved by randomization of preselected patients into the experimental (E) and control (C) groups. The average causal effect of drug E can be estimated by comparing average outcomes across treatment arms (see **Table** 1, 2, 3, 4, 5, 6, 7, 8, 9, 10, 11 for more details). If bias/confounding is minimized by randomization (and ideally combined with double‐blinding), then association can be taken to imply causation, that is, differences between groups in outcome measures can be inferred to be the result of differences in treatment received, not differences in characteristics at baseline or other external influences. This is the “R” in RCT.

The (double‐blind) RCT became the standard^17^ for demonstrating efficacy in the context of marketing authorizations and reimbursement decisions on drugs: a clinically relevant and statistically significant difference between an experimental group and a concurrent comparator group has to be shown. Thus, “difference showing” in an RCT became almost synonymous with estimation of the counterfactual and became the basis of the evidence‐based medicine (EBM) movement.^18^

However, there have always been scenarios in which the counterfactual is sufficiently well understood to obviate the need for an RCT. Such cases are often referred to as “parachute cases,” based on a satirical essay^19^ that pointed out that the use of parachutes has never been subject to an RCT; therefore, according to EBM standards, the efficacy of parachutes should not be trusted even though we know very well what happens if you jump from an airplane without a parachute.

Here, we define a “parachute case” as a situation in which the factual and the counterfactual are sufficiently well understood and in which the difference between the two is likely to be sufficiently large to reasonably exclude chance or bias as an explanation.

We submit that, in the future, assessors of medicines will often have to ask (a) when is the counterfactual sufficiently clear to allow robust inferences about the causal effects of a new treatment (the factual) when an RCT is not feasible? and/or (b) how can we make the counterfactual sufficiently clear, not just for obvious parachute cases?

## Current routes to estimating the counterfactual

We summarize options available for estimating the counterfactual in an order of decreasing robustness but increasing strength of required assumptions.

The (double‐blind) superiority showing RCT is the most widely used study type in the clinical development of new medicines^20^ and generally considered to have the highest level of internal validity.^1^ With a conventional (frequentist) superiority showing RCT, any preexisting knowledge about the counterfactual is deliberately excluded from the statistical analysis, as only analyses based on the randomization will inherit the benefits of randomization and allow causation to be deduced from association. (A special case of the “difference showing” paradigm is the noninferiority [NI] RCT, which is not considered here.)

In a historically controlled trial, the outcome of a group of patients receiving the test treatment is compared to that of a defined group of patients external to the study, which serves as the counterfactual.

With uncontrolled trials, background knowledge (e.g., from the scientific literature) is deemed sufficiently clear to allow inferences about new treatments in the absence of any defined control group, the assumption of natural disease progression serves as the counterfactual. Although ICH E10 expresses caveats about such designs that are based on assumptions that often cannot be verified,^1^ uncontrolled trials have been in special circumstances the basis of marketing authorizations for a sizable fraction of all of medicines' indications recently authorized.^20^, ^21^

Some may take issue with marketing authorizations not based on the “top level” difference showing RCT. However, this is not evidence of a low standard being set by regulators but is a sign of a changing reality. We discuss these changes below and argue that the fraction of new medicines authorized based on methodologies other than difference showing RCTs is set to grow in the future.

## What has changed since the 1948 Streptomycin trial? Addressing the “C” and “T”

In 1948, the frequentist RCT was not only the best but also, in most instances, the only way to estimate the counterfactual in the context of treatment evaluations. Moreover, an RCT could be performed in the majority of new drug development programs.

Much has changed since 1948. Some developments make the conventional RCT less feasible or relevant in a growing number of development programs and necessitate alternative methods of ascertaining the counterfactual (demand side).

## Demand side

### Ethical concerns

Ethical concerns come to the fore in both epidemic and nonepidemic situations of high unmet need in which the natural history of the disease presents high levels of morbidity and/or mortality. Promising results from animal studies, pharmacokinetic/pharmacodynamic experiments, or other findings can create a perceived loss of equipoise posing ethical dilemmas for randomization^22^ and, often, an unwillingness of patients to participate in trials in which they will not receive the experimental medication. In these situations, patients' tolerance for the risk of the unknowns about the product (in terms of both efficacy and safety) is often heightened by the known high morbidity and/or mortality of their disease. Simon *et al*.^23^ identified a number of scenarios in oncology in which it may not be possible to conduct an RCT at all. In these situations, having an alternative trial design to an RCT that, nonetheless, provides interpretable data would be an improvement over current uncontrolled trials and numerous n‐of‐1 compassionate care cases.

### The rise of one‐time interventions with long‐term outcomes

Over the next decade, a growing fraction of new medicinal products will likely be gene therapies, cell therapies, or tissue engineered products.^24^, ^25^ These products come with their own challenges in regard of evidence generation: some of them are expected to be administered only once in a lifetime but the effect size can only be ascertained after prolonged periods.

In some cases, ethical considerations, as discussed above, may preclude RCTs due to the absence of a comparator treatment, perceived loss of equipoise, and high‐unmet need. In other instances, an RCT with an untreated control group may be acceptable, but blinding of patients and clinicians in an RCT is not a realistic option. This may be a result of the need for ancillary interventions, such as myeloablative conditioning^26^ or invasive tissue biopsies.^27^ In such unblinded long‐term RCTs, dropout of patients assigned to the control arm (and crossover to the experimental group, if allowed by the protocol) is expected to be high and will threaten the reliability of results.^23^ An example of the difficulties of conducting RCTs for such therapies was Holoclar, the first cell‐based therapy authorized in the EU.^27^

### Smaller treatment‐eligible populations

A growing number of drug development programs are targeted toward small populations,^28^ diminishing the number of patients available for clinical drug testing within a reasonable time frame. Unsurprisingly, a large fraction of recently approved drugs for rare conditions were not studied in RCTs.^29^

The trend toward the ever smaller treatment‐eligible populations will be accelerated by the quest for precision/stratified medicine.^23^ Comprehensive molecular diagnostic profiling of patients will enable “treatment matching” pathways, in which an individual patient's genomic/phenotypic profile can be matched to a specific treatment. This may yield larger effect sizes but further reduce the practicalities of conducting conventionally powered RCTs in a reasonable timeframe.^30^

### Personalized treatment combinations

There is growing realization that single drug interventions will not be successful in many pathologies.^31^, ^32^, ^33^, ^34^ Most patients may need some form of combination therapy that will be determined individually, based on panels of clinical and biomarker predictors.

Klauschen *et al*.^32^ demonstrated that often tens of thousands of patients would need to be screened to enable a reasonably powered RCT of a given combination of, say, three mutation‐directed treatments. It follows that with increasing combinatorial complexity, difference showing against a matched, concurrent control group will become impossible. An alternative may be to compare a group of patients with a combination of defined biomarkers (e.g., mutations) to unselected patients receiving standard of care. Although technically feasible, such a comparison is no longer like‐with‐like and is flawed if it cannot be ruled out that the predictive markers are not also prognostic.

### Interindividual variance: shift from noise to the focus of interest

The trend toward precision medicine and personalized combinations dictates that another aspect of evidence generation needs to be reconsidered. We recall that the parallel‐group RCT focuses on group‐to‐group statistical comparisons; this is the “T” in RCT. Under this paradigm, interindividual variance must be considered “noise.” Because a high level of noise will reduce the signal‐to‐noise ratio, it follows that variance should be reduced as much as possible to minimize the type II error rate while retaining manageable sample sizes. Yet, as we better understand the biological basis of variance and its implications for therapy selection (e.g., because of multiple different sets of mutations), the very notion of variance changes, from being a nuisance to be minimized, variance becomes the focus of research. The research question changes radically from: “Is A better than B in a defined group of patients?” to: “Given that compound A has been convincingly shown to modulate target X (i.e., it has shown pharmacodynamic activity), (how) can we identify patients who will benefit from A, rather than B?” Some would argue that the question could be addressed by way of an RCT subgroup analysis. This may be true for binary or high frequency predictive markers but any realistic RCTs will be hopelessly underpowered to address the combinatorial complexity presented by large numbers of low frequency markers. Klauschen *et al*.^32^ argue “that the requirement of the classical clinical trials that patients should be similar and groups should be homogeneous […] is irreconcilable with the molecular diversity and diverse therapeutic options of the future personalized medicine approaches […].” It is likely that there will be shifts in the judgment of the scientific community whether RCTs are the appropriate standard for development of drugs for diseases with high patient heterogeneity, once the big data concept has effectively entered practice.

To summarize, the scenarios described above converge to make the RCT less practical in a small, but growing fraction of drug development programs; alternative methodologies are needed. Fortunately, promising developments on the “supply side” may enable ever more accurate and precise ascertainment of the counterfactual as an alternative to the RCT.

## Supply side

### Availability of patient‐level RCT data

The recent decade saw lively debate about public availability and sharing of data from completed RCTs. As a result, the growing availability to the research community of individual patient level data from RCTs has already been put to use^35^ and offers scientists a novel opportunity to understand the counterfactual in a given clinical condition. Several pharmaceutical companies have set up procedures to enable researchers to access the patient level data from completed RCTs.^36^, ^37^

Arguably, the largest repository of accessible RCT data on cancers is Project Data Sphere (PDS), an independent collaborative initiative that collects and makes available in analyzable format patient‐level, comparator arm, and phase III cancer data. All data to be shared have been deidentified to ensure patient privacy. PDS aims for 100,000 patients in the near future, a catalyst to foster an open ecosystem in medical research. An illustrative example of the usefulness of PDS data was provided by the reanalysis of data from the control arms of two phase III RCTs, comparing survival and toxicity of two different drug regimens.^38^ The high granularity of the datasets allowed the researchers to control for a large range of covariates.

A similar initiative is the UK‐based Virtual International Stroke Trials Archive (VISTA) that currently holds stroke trial data on >82,000 patients.^39^

Data from PDS, VISTA, and a growing number of other sources offer the advantage of being high quality and, frequently, having been vetted by regulatory agencies in the recent past. The datasets are also often large (i.e., providing for precision of estimates) and have information on a substantial number of covariates (i.e., providing in‐depth understanding of the patient population). As they originate from RCTs, the data may not be of high external validity but can be useful to provide the counterfactual to a cohort of patients treated with an experimental therapy (i.e., a virtual control group).

### Availability of real world data

The quantity of electronic (e) medical data is expanding rapidly. E‐data come from many different healthcare environments and from a range of sources, including administrative insurance claims data, e‐health records, and dedicated registries. There are practical and methodologic challenges in using real world data (RWD) for secondary analyses; these relate to patient consent, ownership of data, protection of personal information, and biases introduced as part of the data‐capture process.

Nonetheless, there are now initiatives with sufficient time‐in‐use to demonstrate the potential of querying RWD to answer both effectiveness and safety questions about particular treatments. Both distributed (e.g., Sentinel, ENCePP) and centralized data systems (e.g., Optum Labs) have been useful for rapidly exploiting the wealth of information that, at present, remains largely hidden in numerous isolated and scattered healthcare environments.

Similar to extant patient‐level RCT data, this information can be used to construct cohorts of patients satisfying predefined selection criteria to ascertain their outcomes over time and natural history of disease, that is to estimate the counterfactual for a given treatment scenario. Advantages in using RWD to estimate the counterfactual include: (i) high external validity, in which the data are taken not from an artificial RCT situation but from daily clinical practice (note that this can also increase the chance of bias when comparing RWD to data generated in an experimental setting); (ii) multisourced information, in which the data can be gleaned simultaneously from different healthcare environments providing an opportunity to assess reproducibility; (iii) speed and relevance, in which the turnaround time from formulating the research question to obtaining results from RWD can be as short as a few months. This is of importance as the proximity in time between estimating the counterfactual and the effect of the treatment under study can minimize the distorting influence of “drift.”

On the downside, RWD are usually messier than RCT data; there is often limited data standardization due to differently defined variables, time points for measurements, exposure and event definition, different coding systems, missing data, and lack of information on, for example, patient‐reported outcomes.

RWD can even be linked to tissue samples. For example, the Life Raft Group (LRG) has set up a registry matching clinical data of patients with gastrointestinal stromal tumors (GISTs) to tissue samples, donated by patients for the explicit purpose of benefiting all GIST research laboratories worldwide.^40^ The European Organization for Research and Treatment of Cancer (EORTC) has successfully set up similar initiatives for a broader spectrum of cancers.^41^ Observatoire français de la sclérose en plaques (OFSEP), a French registry of more than 40,000 patients with multiple sclerosis, combines clinical data with biological samples and imaging data. Linking archived biologic samples to either RCT data or RWD enables the retrospective determination of (genomic) patient markers long after an observation was made and, with appropriate consent, even after a patient has died.

We consider individual level RCT data and RWD as complementary sources of information that may inform estimation of the counterfactual in a given therapeutic situation. In clinical conditions for which there are both extant RCT data and RWD, the use of both sources can enable data triangulation; information is collected using a variety of methods, with a view to providing more robust estimates than would be possible based on only one single data type.

The European Medical Information Framework (EMIF) initiative integrates large‐scale RWD records with smaller scale, in‐depth translational medicine boutique studies, creating an integrated medical information framework enabling a model‐based approach to answering research questions.^42^

## Progress in methods to analyze non‐RCT studies

We conclude that the “C” in RCT—establishing a control group to estimate the counterfactual—can now be achieved by other means than the concurrent RCT methodology. However, having “some” information about the counterfactual does not in itself assure that like is compared with like. We acknowledge that the “R”—randomization to minimize confounding and bias—is more challenging to replace for the following reasons.

When using historical controls from RWD or external RCT data to estimate the counterfactual to the findings from a cohort of patients treated with an experimental therapy, bias could be caused by confounding (for example, by indication, severity, or prognosis) or a raft of other data issues that threaten the validity of nonrandomized comparisons (see **Table** 1).^2^, ^3^, ^4^, ^5^, ^6^, ^7^, ^8^, ^9^, ^10^, ^11^ However, recent methodological developments in the field of epidemiology and more broadly RWD analyses might help address these issues. Potentially relevant approaches include methods that exploit the concepts of causal inference^12^ to control for confounding (for example, multivariable regression model adjusting for confounders,^43^, ^44^ weighting or stratifying analyses by propensity scores derived from high dimensional covariate data^6^, ^7^, ^8^, ^9^) or using between‐provider variation in prescribing preference as an instrument in an instrumental variable analysis^11^ (for more details on statistical methods see **Table** 1).^2^, ^3^, ^4^, ^5^, ^6^, ^7^, ^8^, ^9^, ^10^, ^11^

These methods may be useful as sensitivity analyses to the primary direct comparison of average outcomes among contemporary and historical patients. However, it should be noted that some causal approaches rely on strong, untestable assumptions and can be highly sensitive to departures from these. Thus, when interpreting results, the analyst must bear in mind that the differences between the primary and causal sensitivity analyses may be due to departures from assumptions rather than confounding.

Nonetheless, these and other methodological developments as well as improvements in quality and interoperability of data may bring the research community closer to accepting the validity of these improved historical controls.

## Design and analysis of Bayesian RCTs

Bayesian RCTs begin with a thorough evaluation of what is understood about trial treatments, once new data become available, this prior understanding is updated using Bayes theorem to make posterior inferences about treatment effects.^45^, ^46^ Individual patient data from completed RCTs and RWD provide a rich source of information that can be mined to formulate prior distributions in Bayesian RCTs. The increasing availability of these data has led several authors^47^, ^48^, ^49^ to propose the concept of “borrowing strength” from existing patient‐level or summary data to augment contemporary information on the control arm (i.e., the counterfactual) of an RCT. “Borrowing strength” is most easily performed in a Bayesian framework and could allow for a more efficient allocation of trial resources to the test treatment because fewer patients need to be randomized to the control group. Some companies may use Bayesian techniques to incorporate historical data into phase II studies and to inform internal decision‐making regarding whether to initiate phase III trials.^50^ However, we are unaware of any drug products authorized based on pivotal RCTs with borrowed data. The approach may gain more traction in the future, as data from past clinical trials are shared more widely. We consider the concept of borrowing strength as a precursor to the threshold approach.

## Threshold‐crossing, description of the approach

A new framework for evidence generation will be required for select drug development programs to address the challenges described above; we propose an approach we call “threshold‐crossing.”

The concept capitalizes on the growing availability of pertinent patient data and is based on an idea that is cursorily mentioned, but not further elaborated on, in the ICH E10 guideline: “It may be tempting in exceptional cases to initiate an externally controlled trial, hoping for a convincingly dramatic effect, with a prompt switch to randomized trials if this does not materialize.”^1^

Our ideas are, in part, building on what was termed a multistage approach, proposed for Ebola treatments by Cooper *et al*.^51^ Elements of the threshold‐crossing concept have been proposed and used in the context of phase II oncology studies (e.g., the Simon design for phase II).^52^, ^53^ Many single‐arm studies in oncology have a prespecified response threshold. They are similar in spirit as both are single‐arm trials and are looking to detect large effect sizes. In both cases, even in the light of potential biases, the evidence‐supporting efficacy of the drug should be unequivocal. There are also similarities between our proposed concept and the establishment of a NI margin in the context of NI trials. In such trials, an NI margin has to be predetermined before the trials starts, ideally derived from a systematic review of preexisting studies, usually placebo‐controlled RCTs.

Threshold‐crossing involves upfront definition of an appropriate estimand defining in detail what needs to be estimated to address the scientific question of interest, based on the treatment‐eligible population, the variable of interest, and the measure of intervention effect.^54^, ^55^, ^56^, ^57^ Subsequently, the counterfactual is determined in a transparent way from existing RWD and/or past RCT data (e.g., some performance measure in a defined population that had been on currently available treatment) and an efficacy threshold higher than the estimate of the counterfactual is set and agreed by relevant decision‐makers alongside a detailed study protocol and analysis plan. Only then will patients who conform to the predefined treatment eligibility criteria be enrolled in a single arm study. In case the threshold is crossed, efficacy is judged to be established. If the result is inconclusive, the product is channeled to a conventional RCT (where practical) or to a second single arm study (in case an RCT cannot be performed). A poor result will lead to prompt termination of the product development.

Note that our discussion is focused on demonstration of efficacy. Applying the threshold‐crossing concept to demonstration of safety will be more challenging because safety is usually not measured by a single variable.

**Figure 1:**
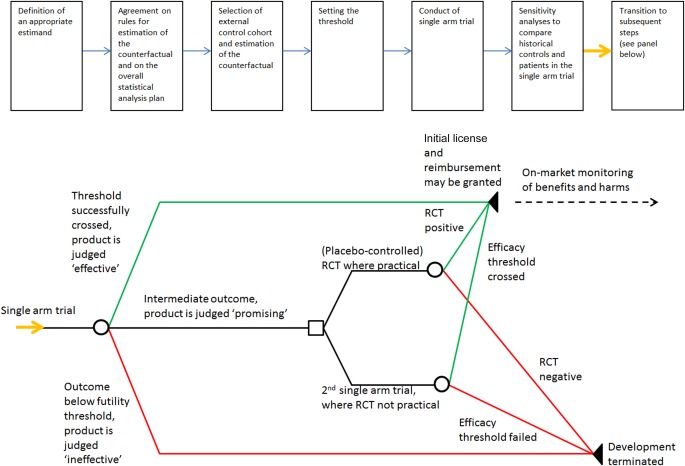
Flow diagram of a threshold crossing trial. The top panel shows the initial, linear sequence of steps, and the bottom panel describes the adaptive follow‐up after completion of the initial single‐arm trial. RCT, randomized controlled trial.

A flow diagram of the threshold‐crossing process is shown in **Figure** 1. In the following, we describe the individual steps in more detail.

### Step 1. Definition of an appropriate estimand

The first step in a threshold‐crossing trial is a precise definition of the estimand, starting with the treatment‐eligible population. Normally, this will be the population described by phenotypic and genotypic criteria that is expected to derive the greatest clinical benefit from the investigational treatment. There is debate whether clinical trial populations should be narrowly or broadly defined. These considerations apply equally to conventional RCTs and to threshold‐crossing trials and will not be further elaborated on here. However, it bears emphasizing that selection criteria need to be sufficiently precise to allow for unequivocal definition of the historical control cohort and the contemporary intervention cohort (see below).

Precise definition of the variable of interest is essential to agree on what is measured and how. For example, a given clinical outcome could be based on a range of clinical scales, assessed at various time points, and expressed as relative or absolute difference, or by way of responder definition. Hence, an upfront agreement on a precise definition of estimand is critical to avoid post‐hoc cherry picking.

Finally, the measure of intervention effect quantifies the treatment benefit in terms of the variable(s) of interest. It should adjust for covariates that are relevant for describing the treatment benefit. It should also address, in a way that is consistent with the scientific question of interest, the impact of treatment‐related events occurring after initiation of study treatment, such as noncompliance, discontinuation of intervention, treatment switching, or use of rescue medication.

For additional considerations on how to select estimands in clinical practice, we refer to the forthcoming addendum of the ICH E9 guidance.^55^

### Step 2. Agreement on rules for estimation of the counterfactual and on the overall statistical analysis plan

Rules for estimation of the counterfactual for the chosen estimand have to be established before selection of the historical cohort. This step is analogous to drafting the statistical analysis plan of an RCT, and should be pre‐agreed with regulators and other decision‐makers, where applicable. The statistical analysis plan should also provide details of the transition to subsequent steps (as described under step 7 below).

### Step 3. Selection of historical control cohort and estimation of the counterfactual

Following the definition of selection criteria (step 1); one or more suitable control cohorts are identified from RWD, existing RCT data, or a combination of both. Normally, patients in the control cohort will have received standard of care, best supportive care or similar, perhaps with add‐on placebo administration if the control cohort is extracted from past RCT control arms. Sufficient and robust information must be available from the data sources to enable the estimation of the average counterfactual outcome for the prospective single‐arm trial.

This step is susceptible to bias. Cherry picking of a “favorable” historical control group (i.e., selection of controls in which the outcome/effect of comparator treatment is artificially poor) is a threat to the internal validity of trial results. As selection bias cannot be controlled for at the analysis stage, safeguards should be put in place at the design stage to minimize the risk of its occurrence. (i) Historical control groups should be identified from a systematic, transparent, and reproducible review of the existing evidence following a prespecified protocol. Methods and guidelines for the conduct and reporting of systematic reviews developed in the context of meta‐analyses may be applicable.^58^, ^59^ (ii) Wherever possible, more than one control cohort should be selected from different sources. Multiple sources will provide more heterogeneity of patient populations and treatment settings and enable researchers and assessors to perform sensitivity analysis of the counterfactual. (iii) The control group(s) must be established before patients are enrolled in the prospective, single‐arm trial of the experimental treatment, and agreement be sought with regulators and other decision‐makers.

After the control cohort(s) have been established, the counterfactual and measures of imprecision are estimated by quantifying the historical/external information, according to the overall analysis plan (step 2), along with sensitivity analyses, where feasible.

### Step 4. Setting the threshold

Once the counterfactual has been estimated, a threshold value is determined based on the historical data and agreed with regulators and other decision‐makers. The threshold will be the benchmark for the primary analysis, for example, if the (confidence bounds of the) result from the single‐arm trial (the factual) exceeds the threshold, the treatment will be deemed efficacious.

The motivation for prespecifying the threshold is to avoid the temptation of setting it once the results from the interventional part of the study are known. Thus, the threshold is determined only by historical control data and expert knowledge. If new external control data become available after initiating the single‐arm trial, this information should still be used for further adjusted sensitivity analyses, for example, to assess whether there is a drift in the pattern of response on control.

In addition to the efficacy threshold, a sponsor may wish to also set a futility threshold.^52^ If the result from the single‐arm trial falls below the threshold, the sponsor would terminate the drug development program.

### Step 5. Conduct of single‐arm trial

The interventional phase of a threshold‐crossing trial is no different from the conduct of a conventional single‐arm trial. All patients receive the experimental treatment according to trial protocol. Care must be taken that the experimental group is selected according to the same criteria as the historical control group(s). Concealed allocation is not possible in a single‐arm trial, making it susceptible to several sources of bias (e.g., assessment bias). Although this concern cannot be entirely eliminated, some forms of bias can be mitigated by, for example, blinding assessors to endpoints, such as radiologists assessing tumor response rates.

### Step 6. Sensitivity analyses to compare historical controls and contemporary patients in the single‐arm trial

Ensuring that patients in the contemporary intervention and historical control groups are recruited on the basis of identical selection criteria will help reduce bias but is no guarantee that the groups will be comparable. In addition to the primary analysis, therefore, further sensitivity analyses should be performed to verify the robustness of conclusions and ensure comparability. However, many methods rely on untestable assumptions. The impact of these assumptions on the validity of the final results as well as the impact of unknown and/or unmeasured confounders should be acknowledged.

### Step 7. Transition to subsequent steps

Subsequent steps will be determined by the outcome and in accordance with the pre‐agreed action plan (**Figure** 1). In case the efficacy threshold is successfully crossed, the product is deemed effective. In the absence of prohibitive risks, it can be granted an initial license and, potentially, reimbursement—usually with a pre‐agreed plan for continuing on‐market evidence generation. In case the efficacy result is below a (predefined) futility threshold, the product is deemed ineffective and development is terminated. When the result falls between the two thresholds, the product is considered promising^51^ and the subsequent step will depend on practical considerations: a conventional RCT is performed where practical. Any ethical concerns, as described above, should at this stage be less of an obstacle to performing a placebo‐controlled RCT because the result of the single‐arm trial make a dramatic effect of the experimental treatment unlikely, moving expectations closer to clinical equipoise. However, in some clinical scenarios, an RCT would not be practical, in which case, the trial will be rolled over into a second single‐arm trial, based on an agreed protocol and analysis plan.^51^

## Should the threshold be set high or low?

As discussed above, after estimation of the counterfactual, an efficacy threshold will need to be set some distance above this point estimate (step 4, above). It is evident that the distance drives the risk of false conclusions from the trial results: a large distance between the estimate and the threshold (that is, a high hurdle) will entail a small risk of a false‐positive conclusion (type I error), but a high risk of a false‐negative conclusion (type II error) about the drug's efficacy. Conversely, a low hurdle will entail a high false‐positive but low false‐negative risk. Patients and other stakeholders, such as healthcare payers, will pay a price for either false conclusion.

It is unlikely that there will emerge any hard and fast rules for this critical and potentially controversial step in the threshold approach. However, we submit that the distance between the counterfactual estimate and the efficacy threshold should be determined based on two sets of considerations:

(1) Methodological considerations

Perceived accuracy and precision of the counterfactual: the level of confidence in the validity of the counterfactual estimate will be directly related to (i) the quality and completeness of the dataset(s) from which it was derived, (ii) the total number of patients on whose data the estimate was based, (iii) the number of sources that were available for the exercise, and (iv) the degree of agreement of the estimates derived from the different sources. In turn, the degree of confidence in the robustness of the positive result of the single‐arm trial will be inversely related to the distance between the counterfactual estimate and the efficacy threshold that decision‐makers might be willing to accept.

Potential for bias: even a high level of confidence in the counterfactual estimate is not, in itself, sufficient to ensure credibility of the threshold‐crossing trial. A second *sine qua non* is comparability of the counterfactual to the factual derived from the single‐arm interventional cohort. To enable assessors of the study to conclude that indeed “like‐with‐like” was compared, it will be necessary to understand as many relevant covariates (and their distribution in the historical control and contemporary intervention cohorts) as possible, and to perform additional adjusted sensitivity analyses, as described in step 6 above.

By definition, the presence of unknown confounders can never be excluded in nonrandomized studies, not even in an RCT. With the availability of ever more patient‐level data, and more information on covariates, the risk of unknown confounders in threshold‐crossing trials will diminish. Nonetheless, experimenters and decision‐makers will have to evaluate on a case‐by‐case basis the risk of confounding and bias. In turn, this will inform the choice of the threshold, above which a “parachute case” can be assumed.

(2) Ethical considerations

Montazerhodjat and Lo^60^ have recently argued that the weight given by regulators to avoiding a type I error (approving ineffective therapies) vs. avoiding a type II error (rejecting effective therapies) should not be equal across disease and clinical indications: “For terminal illnesses where patients have no choice but death [like patients with stage IV pancreatic cancer], the relative costs of type I and II errors are very different than for non‐life‐threatening conditions.” They propose RCT designs in which the error rates are informed by some measure of disease burden rather than by “arbitrary” convention (usually α = 2.5% for one‐sided tests, and usually 1 ‐ β = 80%). We envisage that the setting of the efficacy threshold should be influenced by nonmethodological externalities, including severity of disease and unmet need of the target population, availability of alternative treatments, patients' input on what would be clinically relevant to them, societal burden of disease, and expected frequency of serious adverse effects.

## Benefits and risks of the threshold concept

The concept of threshold‐crossing may enable the development of products that are potentially beneficial but difficult to assess in RCTs for the reasons described above. Compared to performing an uncontrolled study and hoping for the best, threshold‐crossing can potentially increase the predictability of, and public trust in, decision‐making about difficult products and indications. This requires that all steps in the process, including the reasons and assumptions for setting the efficacy threshold, are prespecified and fully transparent to decision‐makers, and made public once a product is authorized and reimbursed.

As was described above, an RCT may not be feasible because of a lack of suitable patients available to populate both an experimental and a concurrent control group. However, many more patients, including an accumulating number of deceased patients, will be available in (archived) RWD and RCT databases to provide information on the counterfactual.

*A priori* definition of the success criterion for a single‐arm trial can inform a sponsor's go/no go decision‐making during the early clinical development phase. Once a threshold has been agreed upon with other stakeholders, the decision is only dependent on the sponsor's confidence in the potential of their product and regulatory or HTA/payer uncertainty should be reduced. However, the latter uncertainty can never be entirely eliminated because unexpected adverse effects will impact the final assessment of the product's benefit‐risk tradeoff, relative effectiveness, and value.

With threshold‐crossing, the speed, investment, and effort required up to the point of initial licensing and reimbursement or termination is biased in favor of products that are either highly effective or (near‐)ineffective. This is welcome because it is more important to provide timely access to highly beneficial than incremental treatments, and to terminate quickly and economically nonviable assets.

For the same reason, threshold‐crossing may provide an opportunity to steer pharmaceutical research and development to areas of greater unmet need, as the threshold is expected to be set less stringent (i.e., easier to cross) in such conditions. This would not only address past criticism of pharmaceutical research and development for focusing too many resources on “me‐too” products but may also fast‐track research programs on subpopulations with unmet need ahead of programs for undifferentiated blockbuster indications.^61^

Thresholds make explicit and quantify the currently ill‐defined concept of clinical relevance by declaring upfront what each decision‐maker would consider an effect size of sufficient importance.

Reuse of existing data makes drug development faster and more economical. The majority of currently performed RCTs are balanced two‐arm trials (i.e., patients are randomized 1:1 to experimental and control groups). Obviating the need for a control group could reduce the trial sample size by almost 75% in the best case when a large number of historical controls are available, at least for highly effective therapies. In addition, eliminating the control group will help focus research on those aspects we know least about. If the average response on control is already well characterized, usually, we only seek to learn more about the experimental treatment. Hence, the number of patients needed for the control arm in the parallel group design could be used to increase the number of patients treated with the experimental treatment in a single‐arm trial.

The threshold‐crossing approach addresses the ethical issues associated with RCTs in which prior assumptions about a drug's potential are strong, leading to a (perceived) lack of equipoise. Here, the result from the initial single‐arm trial may render an RCT acceptable.

Most patients enrolling in clinical trials do so with an assumption of contributing to medical knowledge. Reuse of past RCT data to determine the counterfactual to responses on future treatments allow patients to maximize the impact of their philanthropy because more than one research question can be addressed as a result of their participation in a trial.

Although these benefits of threshold‐crossing seem self‐evident, we identify at least two broad areas of risk:

(1) Methodological risk—comparing like‐with‐like

**Figure 2:**
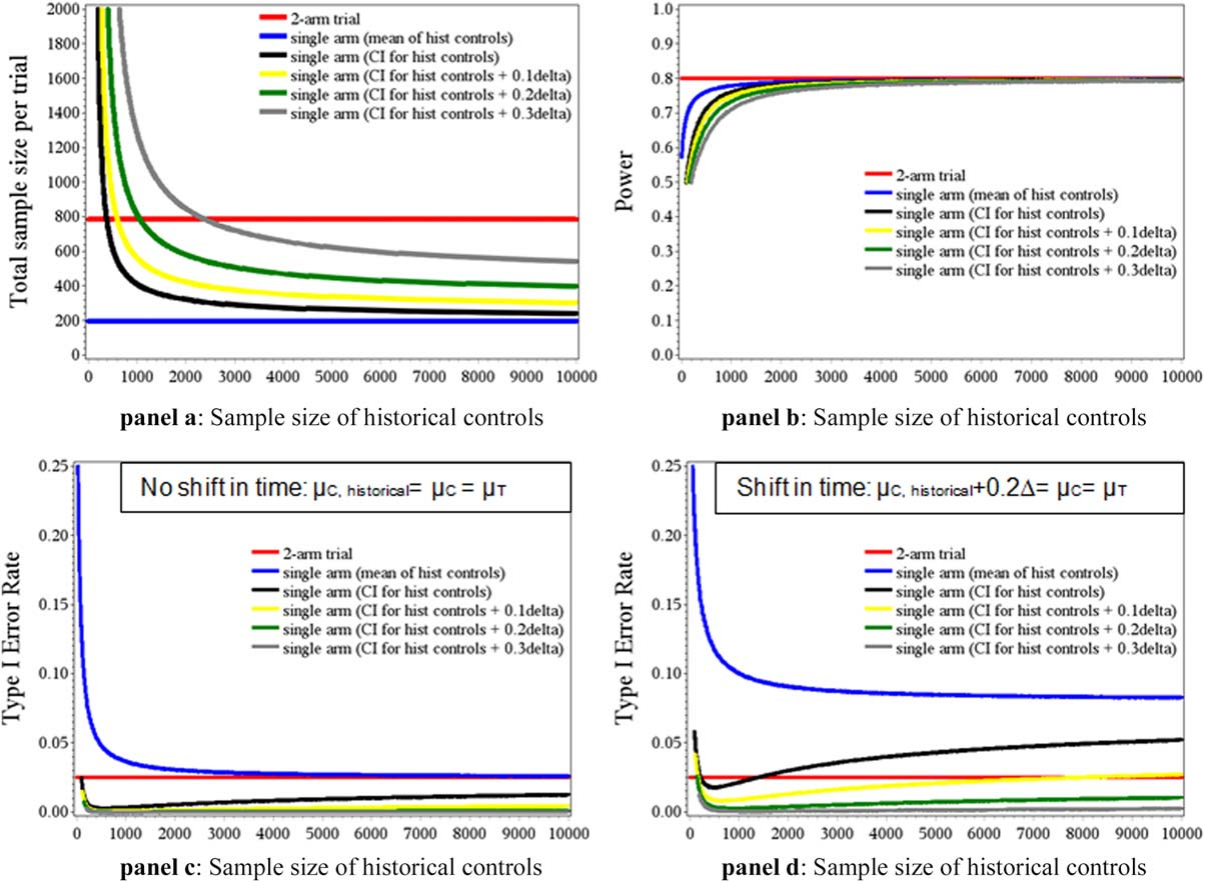
We performed clinical trial simulations to evaluate the operating characteristics of threshold‐crossing trials when frequentist hypothesis tests and corresponding sample size calculations for single‐arm trials are naively applied. To demonstrate the efficacy of a new drug, the most common approach is to conduct parallel group trials to show superiority of the new treatment over control, i.e. testing the null hypothesis $H_{0}:\;\mu_{N}\leq \mu_{C}$ versus the alternative $H_{1}:\;\mu_{N}>\mu_{C}$ at one‐sided significance level of 2.5%, where $\mu_{N}$ and $\mu_{C}$ denote the expected response in the new and control treatment arm, respectively. For the results presented we assume a normally distributed endpoint with σ=1. For example, if such a trial was powered at 80% to detect a standardized effect difference of Δ= $\frac{\mu_{N}-\mu_{C}}{\sigma }$=0.2 between the new and the control treatment, a sample size of around 400 patients per group would be required resulting in a total trial sample size of 800 (red horizontal line in panel a). Alternatively, one may apply a threshold‐crossing single arm trial testing $H_{0}^{t}:\;\mu_{N}\leq t$ versus $H_{1}^{t}:\;\mu_{N}>t$ using a one‐sample test at one‐sided level 2.5%, where *t* is the a‐priori fixed threshold determined from historical controls. What is the impact on the error rates, if one takes a rejection of $H_{0}^{t}:\;\mu_{N}\leq t$ naively as a rejection for $H_{0}:\;\mu_{N}\leq \mu_{C}$? Assume trialists naively use the *observed* mean estimated from historical controls as threshold *t*. A conventional sample size calculation for a single arm trial yields a trial sample size of about 200 for a standardized effect of Δ=0.2. Hence, in a best‐case scenario, with no uncertainty on the effect size in the control arm, sample size can be reduced to a quarter relative to a parallel group design. However, due to sampling variability, the observed mean in the controls typically does not coincide with the true population mean $\mu_{C}$ (even assuming $\mu_{C}$ would be identical for historical and concurrent controls). As a consequence, the power to reject $H_{0}$ decreases with decreasing sample size in the historical controls due to increasing variability of the historical estimate (blue line panel b). In addition, the type I error rate to erroneously reject $H_{0}$ can be substantially inflated for small sample sizes of historical controls (blue line panel c). In contrast, both the type I error rate and the power (if the true standardized effect is indeed Δ=0.2) of the parallel group design with concurrent controls do not depend on the historical data (red line in panels b and c). The uncertainty due to the sampling variability when estimating the historical response could be addressed by a more cautious choice of the threshold t, e.g., taking the upper boundary of a two‐sided 95%‐confidence interval for *µ_C_* computed from historical controls. A conventional sample size calculation for a single arm trial accounting for a higher threshold (i.e., adjusting the standardized effect 0.2 size by the half width of the confidence interval) yields a sample size of about 400 (=half of that for the parallel group design), if about 1000 historical controls were available (see black line in panel a). The more historical data are available, the lower the resulting sample size for the new threshold‐crossing trial. Assuming $\mu_{C}$ is identical for historical and concurrent controls, the type I error rate is controlled (black line panel c), however a loss of power is observed if the historical control data base is small (black line panel b). Furthermore, if $\mu_{C}$ differs between historical and concurrent controls, e.g., the mean response under control treatment is increasing over time, there might be an inflation of the type I error rate with the thresholding single‐arm design (panel d black line), but not for the traditional two arm parallel group design (with concurrent controls). To address such biases, one may apply even more conservative (larger) thresholds t, for example by adding a percentage of the assumed standardized effect to the upper boundary of the historical 95% confidence interval (e.g., adding 0.1Δ, 0.2Δ, and 0.3Δ for yellow, green and gray lines in panels). This comes at the cost of larger sample sizes (see panel a), but by using sufficiently conservative (large) thresholds, an inflation of the type I error rate to erroneously reject $H_{0}$ can be avoided (see green and gray line in panel d). For simplicity we have assumed that all historical controls come from one data source, e.g., a single clinical trial or a registry. If several sources are to be used, one has to account for between trial variability as well, e.g., by replacing the sample mean estimate of *µ_C_* by a meta‐analytic estimate of *µ_C_* obtained from a fixed or random effects meta‐analysis of historical controls. panel a: Sample sizes, power and type I error rate are given for a parallel‐group design and single‐arm threshold designs applying different thresholds. The sample size of the historical controls is shown on the x‐axis. The operating characteristics of the designs shown in panels b, c, and d are based on the sample sizes shown in panel a (that depend on the size of historical controls and assumed thresholds).

The obvious advantages of the RCT over other methods of estimating causal effects are randomization and blinding; these features provide the best known way (but no guarantee) of reducing bias and imbalances between treatment groups due to known or unknown confounders. By contrast, the threshold‐crossing concept does not provide these safeguards and is at higher risk of comparing like‐with‐unlike, potentially leading to false conclusions. For example, **Figure** 2 shows that there might be a large inflation of the type I error for the threshold design, but not for a standard two‐arm RCT, if the treatment effect is changing over time.

Therefore, we are not advocating that threshold‐crossing trials should replace RCTs but believe they should be explored as a pathway for selected clinical development programs. We are confident that with increasing availability of better and more granular patient data and with growing experience in analyzing such studies, any sweeping arguments about “the inherent lack of internal validity of nonrandomized studies” will lose ground.

(2) Expectation risk—setting (un)realistic thresholds?

We have argued that a threshold‐crossing trial increases decision‐maker predictability compared with conventional developments. However, this is only true from the moment when a threshold has been agreed on. It could be argued that regulators, HTA bodies, and payers will insist upon unrealistically high thresholds to guard against any false‐positive decision. This risk could be mitigated by ensuring that patients are involved in a systematic and transparent way when the threshold is set and that their voice is heard on what is clinically relevant to them.

The proposed framework focuses on efficacy. In practice, however, the threshold‐crossing approach will have to be implemented with a view to demonstrate an acceptable benefit‐risk profile. Safety and tolerability are usually not measured by a single variable and are often associated with low incidences. Hence, the formal framework will not likely be applicable for safety assessment. It bears reminding, though, that a formal evaluation plan is also not part of the difference‐showing paradigm, at least in cases of small RCTs. Under any paradigm, safety assessment has to follow the more conventional approach of “looking at the totality of data.”

As for any new drug, a plan for postlicensing knowledge generation should be in place at the time of initial licensing, describing methods and milestones for studies to generate additional affirmative evidence on efficacy and safety.

## The way forward

A range of practical, science, and policy issues need to be addressed before the potential of the threshold‐crossing approach can be exploited.

Here, we provide a gap analysis and recommend concrete steps to enable the conduct of threshold‐crossing trials in the future. (a) Development of a formal set of eligibility criteria. The threshold‐crossing paradigm should be reserved for carefully selected products and clinical conditions in which RCTs are not practical. Hence, agreement needs to be reached on qualifying criteria for candidate assets to be considered justifiable for threshold‐crossing. A starting point for such discussions could be the Bradford Hill considerations on causality^62^ and the criteria proposed by Byar *et al*.^63^ to justify uncontrolled phase III clinical trials. (b) Practicalities of clinical data sharing. Sharing of clinical trial data has come a long way and there is now broad in principle consensus that all data from past RCTs and other sources should be made available to facilitate future research. However, practical issues must be resolved before threshold‐crossing trials can be conducted within a reasonable timeframe and with reasonable effort. This includes, but is not limited to, issues of data format and interoperability, appropriate level of deidentification, governance, and legal interaction between data custodians and data requesters. For sensible interpretation of historical data, access is needed not only to the raw data but also to the clinical trial protocols and statistical analysis plans together with the software code, data dictionaries, and the study reports.^64^ Several groups, such as the UK‐based Wellcome Trust (for past RCT data) or the Sentinel Initiative in the United States and the Farr Institute in the United Kingdom (for RWD) are establishing themselves as leading data custodians or data brokers. We propose that a dedicated project be funded (e.g., by the EU Innovative Medicines Initiative (IMI)), to set global standards and processes for data sharing for virtual control groups. The proposed project would bring together data custodians, drug developers, and prospective trial assessors (regulators, HTA bodies, payers) and would, hopefully, be the last step in the on‐going data‐sharing journey. This project could also address recommendations c and d. (c) Archived biological samples. Knowledge about stratification biomarkers emerges on an ongoing basis, and it is to be expected that information on a (genomic) stratification marker may not have been analyzed in the past in a group of patients that would now be relevant for estimating the counterfactual. In such cases, it will be helpful to have access not only to electronic data archives but also to archived tissue or other biosamples that may still be available (e.g., from past RCTs).^23^ Hence, the pivotal role of biobanks for threshold‐crossing trials must be acknowledged and their establishment and usability supported. This needs to encompass standards for the data collection methods, quality assurance (certified biobanks and annotation of samples), and achieving the right balance between data protection and data availability for researchers. (d) Patient informed consent. Aside from technical and governance bottlenecks to data sharing, a key issue is the question over the need for dedicated patient consent for the reuse of their data (and biosamples, where applicable). This has proved to be an obstacle for some attempts at secondary use of patient data in the past and will require novel legal and political solutions that may be different in various jurisdictions. For the European Union, for example, the new Clinical Trial Regulation^65^ and the new General Data Protection Regulation^66^ have introduced provisions enabling reuse of personal data for the purpose of scientific research. (e) Patient education and involvement. We have outlined that patient involvement will be key to ensuring the success and acceptance of threshold‐crossing trials. If the process of patient involvement is to be representative, transparent, and reproducible, it cannot be based solely on a convenience sample of patients and anecdotal input. Systematic, validated methods and specific patient training will be required to ensure meaningful guidance on what is relevant to patients themselves. A number of groups, including the IMI PREFER consortium, are exploring such methods and we would encourage them to consider adding threshold concept issues to their ongoing projects. (f) Methodology development. Computational techniques used in other fields could be used to run the simulations and develop the virtual control arms needed for the threshold‐crossing concept (see **Figure** 2 on statistical simulations). Well‐established epidemiological analytic methods might be applicable to single arm clinical trials incorporating historical controls to enable causal inferences. Sensitivity analyses will be essential to elaborate on how underlying assumptions impact the interpretation of trial results. Bayesian methods are well suited for informing internal decision‐making in drug companies (e.g., go or no‐go decisions from phase II to III). Similar techniques could be developed to incorporate historical information into regulatory and reimbursement decisions. The uptake of Bayesian methods would be encouraged by more examples of successful applications. Clinical trial simulations to determine the (frequentist) operating characteristics of Bayesian approaches will assume a more prominent role. How to design, perform, and report such simulation studies deserve further attention and standardization. For reproducibility, documented software (code) should be made accessible to all stakeholders. However, if simulations are computationally intensive or the code is complex, it will be challenging to independently verify the results. Quantitative methods to extrapolate from existing information to support decision‐making are addressed in a recent draft reflection paper.^67^ Another important avenue for future research is the development of statistical methods that balance the need to anonymize patient data with the need to preserve essential information. (g) Piloting the threshold approach. Finally, a prudent way to introduce a new concept is to test whether it works in theory before deploying it in practice—to “look before leaping.” We propose using existing platforms that will allow learning about the concept retrospectively before applying it prospectively. This work should include reanalysis of RCTs as single‐arm trials with historical comparators, where possible, to see whether similar results are obtained—and if not, why not. Existing multistakeholder collaborations, like projects sponsored by the IMI or the Massachusetts Institute of Technology‐based NEWDIGS, might lend themselves as potential fora.

In parallel, a compromise between single‐arm trials and two‐arm trials could be “hybrid” designs,^48^, ^50^, ^68^ using unequal randomization and augmenting the control arm with historical data. Adaptive versions of these designs would also offer the flexibility to recruit fewer concurrent controls if interim data supported the commensurability of historical and contemporary patients.^47^ However, adaptive designs have their own caveats that go beyond the scope of this paper.^69^, ^70^

## Conclusions

It is time to make use of a resource that was not available to the RCT pioneers in the mid‐20th century but is now becoming abundant. Rich data on past and current patients can provide much needed information on the counterfactual for emerging treatments.

The benefits of using existing data in the framework of a threshold‐crossing study are self‐evident: fewer patients need to be enrolled in trials and the value of data gleaned from those who do will be multiplied; the efficiency and speed of clinical trials are increased; and clinical research could be nudged toward highly effective treatments.

On the downside, we reiterate that the usefulness of the threshold‐crossing approach will ultimately hinge on the ability to minimize confounding and bias. Larger sample sizes afforded by the use of preexisting data will not, *per se*, address this issue.

We are not advocating replacing RCTs with threshold‐crossing trials but they should be explored for selected development programs that are difficult to run with current methodologies. The practical obstacles that need to be addressed and the conceptual risks of the proposed approach must be fully understood and tested before threshold‐crossing trials become a part of the standard drug development toolbox.

Finally, it bears remembering that any prelicensing evidence, randomized or not, requires confirmation and expansion by way of on‐market evidence generation.

## SOURCE OF FUNDING

This project has received funding from the European Union's Seventh Framework Programme for research, technological development, and demonstration under grant agreement no 603160 (M. Posch: Asterix ‐ Advances in Small Trials dEsign for Regulatory Innovation and eXcellence) and no 602552 (F. König and P. Bauer: IDEAL ‐ Integrated Design and Analysis of small population group trials). Sebastian Schneeweiss is Principal Investigator of the Harvard‐Brigham Drug Safety and Risk Management Research Center funded by FDA. His work is partially funded by grants/contracts from PCORI, FDA, and NIH.

## AUTHOR CONTRIBUTIONS

Hans‐Georg Eichler and Brigitte Bloechl‐Daum contributed equally to this work.

## CONFLICT OF INTEREST

Sebastian Schneeweiss is consultant to WHISCON, LLC and to Aetion, Inc., a software manufacturer of which he owns equity. He is principal investigator of investigator‐initiated grants to the Brigham and Women's Hospital from Genentech and Boehringer Ingelheim unrelated to the topic of this study. Mark Trusheim is President of Co‐Bio Consulting, which provides management consulting services to biomedical firms. Peter Honig is an employee of Pfizer but contributed to this article in his personal capacity only.

## DISCLAIMER

The views expressed in this article are the personal views of the authors and may not be understood or quoted as being made on behalf of or reflecting the position of the agencies or organizations with which the authors are affiliated.
